# Self-directed music play to improve executive function in young children using NIRS

**DOI:** 10.1038/s41598-025-10984-1

**Published:** 2025-07-22

**Authors:** Taku Kosokabe, Makoto Mizusaki, Wakako Nagaoka, Miwa Honda, Noriyuki Suzuki, Yusuke Moriguchi

**Affiliations:** 1 Miyagi University of Education, Sendai, Japan; 2 Tokyo Gakugei University, Koganei, Japan; 3 Hamamatsu Gakuin University Junior College, Shizuoka, Japan; 4 Aichi Gakusen College, Okazaki, Japan; 5 Tokiwa University, Mito, Japan; 6 Graduate School of Letters, Kyoto University, Yoshidahoncho, Kyoto, 606-8501 Japan

**Keywords:** Music play, Executive function, Prefrontal cortex, Preschoolers, Neural efficiency, Functional near-infrared spectroscopy (fNIRS), Psychology, Human behaviour

## Abstract

Play is fundamental to child development, with evidence highlighting its crucial role in enhancing executive function (EF). However, the neural mechanisms underlying play-based cognitive improvements remain poorly understood. Here, we examine how music play influences behavioral performance and prefrontal cortex (PFC) activity in preschoolers using functional near-infrared spectroscopy (fNIRS). In a randomized controlled trial, 57 three-year-olds participated in either an Orff-Schulwerk-based music play program emphasizing creative self-expression (n = 27) or a control program (n = 30). The music play group showed modest improvements in inhibitory control tasks compared to the control group. Notably, fNIRS measurements revealed distinct patterns of neural activation between groups: the control group exhibited increased PFC activation during the post-test, whereas the music play group maintained stable neural activity levels, suggesting enhanced neural efficiency. These findings demonstrate that different types of activities are associated with distinct patterns of behavioral improvement and neural activity changes in young children. These results offer new insights into how play-based interventions shape cognitive and neural development and highlight promising directions for early childhood education programs to foster cognitive development.

**Supplementary Information:**

The online version contains supplementary material available at 10.1038/s41598-025-10984-1.

## Introduction

Executive function (EF) refers to self-regulatory cognitive processes that control thought, action, and emotion, enabling goal-directed behavior. EF includes working memory, inhibition, and cognitive shifting^1–3^. Neuroimaging research has shown that EF relies on a distributed fronto-parietal network^4,5^. The dorsolateral prefrontal cortex (DLPFC) plays a critical role in maintaining and manipulating information as well as in task-switching. The ventrolateral prefrontal cortex (VLPFC) primarily involves response selection, while the posterior parietal cortex contributes to attentional control. These regions form an integrated network that coordinates activity during complex cognitive tasks, with activation patterns shifting developmentally as neural efficiency increases. EF development has been linked to the maturation of the prefrontal cortex (PFC) and its associated neural networks^6,7^. This development is particularly rapid in early childhood and continues to be important throughout childhood and adolescence^1,8^. Moreover, EF plays a fundamental role in children’s overall development, influencing their ability to understand others’ mental states, build peer relationships, succeed academically, and maintain good health and income^9–13^.

Recent studies have explored various methods to improve EF in children, including both direct and indirect training approaches^14,15^. Direct training involves repetitive practice of specific EF tasks, whereas indirect training incorporates activities that engage EF, such as play-based interventions. Researchers suggest that indirect training, particularly when it includes elements of play, may be more effective in transferring benefits to other cognitive domains^14,16^. Play, a fundamental aspect of child development, encompasses various activities, including pretend play, constructive play with blocks and toys, physical play, social play with peers, rule-based games, exploratory play in nature, and creative expression through music^17,18^. Play is intrinsically motivated, and children engage in it for its own sake. Research demonstrates that different types of play contribute to developing language skills, problem-solving abilities, creativity, and social cognition^19–21^.

Regarding EF, Vygotsky^22^ proposed that children improve their self-regulatory behaviors through pretend play. He suggested that the rules inherent in pretend and dramatic play help children act against immediate impulses, thereby strengthening their self-regulatory skills. Moreover, the self-directed nature of play may further support EF development^23^. Unstructured playtime requires children to exercise autonomy in decision-making, regulate their behavior, and purposefully engage in goal-oriented activities that activate EF capabilities. Research has shown a positive relationship between EF development and the amount of unstructured time in children’s daily routines, including spontaneous play^24^.

Music play, a form of play, has garnered attention in EF research. Music involves organizing sound in time and incorporates various elements such as pitch, melody, rhythm, dynamics, and timbre. Its ubiquity in human life and its ability to modulate stress make it a compelling subject in child development research. In this study, “music play” refers specifically to teacher-planned music play activities implemented for the entire class. This differs from spontaneous “musical play,” as described in the literature^25^, which is primarily child-initiated. While music play hare is teacher-planned, they differ fundamentally from didactic instruction, where children merely follow directions or imitate demonstrations. Although structured by teachers, our music play activities prioritize children’s autonomy and creativity, ensuring play remains central to the experience.

Our work builds on Valerio, Reynolds, Bolton, Taggart, and Gordon’s^26^"Music Play," which presents various activities designed to promote music development in young children based on Gordon’s Music Learning Theory^27^. This approach aligns with the self-directed nature of play, which is beneficial for EF development. Several studies have examined the relationship between music play and EF skills, particularly inhibitory control^28–32^. While music has been shown to enhance EF, its effects on inhibitory control are more consistently pronounced than on working memory and cognitive shifting^33^.

Building on these findings, this study focuses on the Orff-Schulwerk approach as a specific form of music play for enhancing EF skills. Developed by Carl Orff in collaboration with Gunild Keetman, this approach emphasizes integrative and creative expressive play through improvisation, where music, movement, and speech are interconnected through rhythmic elements^34,35^. It aligns with play-based EF enhancement theories, incorporating elements of self-directedness, creativity, and multi-modal engagement. A recent study^29^ provided compelling evidence on the effectiveness of the Orff-Schulwerk approach in enhancing EF skills, showing that young children who participated in an Orff-Schulwerk-based program exhibited significant improvements in inhibitory control and working memory compared to a control group.

Its potential to enhance EF and prefrontal activity can be attributed to several factors. First, this approach fosters active engagement with musical elements through exploration, improvisation, and creative expression, aligning with the self-directed nature of play that supports EF. Second, it integrates music, movement, and speech, potentially activating multiple brain regions and strengthening their connections. Finally, it emphasizes group activities, providing rich opportunities for social interaction, which requires real-time coordination, turn-taking, and mutual adjustment—all heavily reliant on EF skills. For example, during group improvisation, children must inhibit impulses to play out of turn (inhibitory control), remember the overall structure of the piece while contributing their part (working memory), and adapt flexibly to changes in rhythm or melody (cognitive shifting). These cognitive demands during social musical interactions may stimulate the prefrontal cortex, potentially leading to enhanced EF development.

Although a previous study^29^ provides valuable insights into the potential benefits of music play on young children’s EF skills, but it was limited to behavioral measures. Therefore, whether play-based interventions truly influence the neural basis of EF development remains unclear. To address this limitation and enhance our understanding of how play affects EF development, this study aimed to investigate the neural correlates of EF before and after music play-based intervention. Specifically, we examined PFC activity, a known neural substrate of EF, using functional near-infrared spectroscopy (fNIRS) both before and after the intervention.

Recent neuroimaging studies have demonstrated that children recruit the PFC during various EF tasks, including inhibitory control^36^, cognitive shifting^37,38^, and working memory tasks^39^, as measured by fNIRS. These findings highlight the importance of examining prefrontal activation patterns to understand the neural mechanisms underlying EF development in young children.

Building on previous behavioral findings^29^, this study investigated whether music play influenced PFC activity in young children. Using a randomized controlled trial design, we investigated the effectiveness of music play on EF skills and PFC activity. We examined group differences in multiple EF measures—inhibitory control, working memory, and cognitive shifting—before and after the intervention. Additionally, we assessed PFC activity during inhibitory control tasks, as previous studies have reported the effectiveness of music interventions on inhibitory control measures^31^.

We compared EF performance and PFC activation between two groups of young children: one group participated in music play programs, while the control group engaged in teacher-directed educational activities without self-guided components, such as group singing under instruction. We hypothesized that participants in the music play program would demonstrate more significant improvements in EF assessments and prefrontal brain activity than those in the control condition.

## Methods

### Participants

Children were recruited from a nursery school in Japan. Of the five classes of 3-year-old children, two classes were assigned to the music play group, and three to the control group. This study included 27 children in the music play group (*M* age = 47.4 months; *SD* = 3.8; range = 41–53 months; 13 girls) and 30 children in the control group (*M* age = 46.4 months; *SD* = 3.7; range = 41–53 months; 15 girls). This sample size is comparable to other neuroimaging studies involving interventions in young children^40^.

Demographic analyses revealed no statistically significant differences in age or gender distribution between the groups (*p*s > 0.312). This study was approved by the Ethics Committee of Miyagi University of Education (protocol number 4) and adhered to the ethical standards established by the Helsinki Declaration of 1964 and its subsequent revisions. All parents provided written informed consent before the study commenced.

#### Intervention program

The music play program was inspired by the Orff-Schulwerk approach, a pioneering concept in music education developed by Carl Orff in collaboration with Gunild Keetman. This approach integrates music and movement through rhythmic, improvisational, and creative activities, fostering a holistic learning experience. Orff-Schulwerk encourages children to explore and express their musical creativity in their own distinctive ways from an early age.

The program was designed based on seminal works in the Orff-Schulwerk tradition, including *Musik für Kinder I-V*^34^ and the updated *Musik und Tanz für Kinder, Unterrichtswerk zur Früherziehung I-II*^41^. These resources provided a strong foundation for developing activities that allowed children to engage in uninhibited creative self-expression through music and movement.

A key strength of this program was its accessibility for educators, regardless of their musical background. To ensure effective implementation, teachers participated in five intensive 90-min workshops, where they were introduced to six distinct units and collaborated with the program coordinator to tailor activities to their specific educational contexts. The program strongly emphasized fostering children’s autonomy and self-directed learning.

The program comprised six core units, each repeated five times over 30 lessons, encompassing various music activities:

- *Name play:* Children experimented with rhythm and pitch, using their names as a basis for call-and-response improvisation.
- *Singing play:* Improvisation was combined with simple percussion instruments, set to the rhythms and melodies of familiar traditional children’s songs.
- *Movement play:* Children were encouraged to create spontaneous movements in response to musical rhythms or guided imagery.
- *Rhythmic play:* Children focused on body percussion, and crafted rhythms using different body parts and mimicking their peers.
- *Instrumental play:* Children explored various percussion instruments through playful experimentation.
- *Drum play:* Children listened to stories, allowed their imaginations to expand, and translated these experiences into rhythmic movements and drumming.

Through these activities, the program aimed to provide a comprehensive musical experience while fostering creativity, self-expression, and cognitive development.

#### Control program

The control group participated in a structured, teacher-led program that did not emphasize self-directed activities. It included:

- *Guided singing:* Children sang pre-selected songs in unison, following teacher instructions. Songs were chosen to align with age-appropriate themes and learning objectives.
- *Literature exposure:* Teachers conducted read-aloud sessions using picture books to enhance listening skills and stimulate imagination through storytelling.

This program maintained a consistent structure across sessions, with educators taking a directive role. Unlike the music play group, it placed limited emphasis on child-initiated activities and creative self-expression.

### Behavioral measures

To assess EF skills, we used a battery of tasks measuring inhibitory control, cognitive shifting, and working memory. Only inhibitory control measures were used for fNIRS recordings. Given our limited testing time and resources, we made strategic decisions based on scientific rationale and feasibility. Preschool children’s attention spans and cooperation levels are limited, making comprehensive fNIRS measurements across multiple tasks impractical. Each additional task increases the risk of data loss due to movement artifacts or participant fatigue. Our focus on inhibitory control for fNIRS measurements was guided by prior research, which has shown that music interventions most significantly impact this EF component^40^.

#### Inhibitory control

We utilized two tasks to evaluate inhibitory control:

- *Black/white task:* Adapted from a previous study’s^42^ procedure, this task required children to suppress a prepotent response. Initially, they pointed to cards matching a verbal cue (e.g., pointing to a white card when prompted “white”). In the test phase, the rule was reversed, requiring them to point to the opposite color (e.g., pointing to a white card when prompted “black”). After practice trials, children completed 10 test trials, with performance measured by the number of correct responses.
- *Hand game:* Based on Luria’s work^43^, this task assessed motor inhibition. Children were instructed to perform a gesture opposite to the experimenter’s (e.g., making a fist when shown a pointed finger). After practice trials, they completed 10 test trials, and accuracy was recorded.

For both tasks, children completed four sessions each for fNIRS measurements, yielding a total possible score of 0–40 points.

#### Cognitive shifting

We assessed cognitive shifting using the Dimensional Change Card Sort (DCCS)^44^. Children sorted cards according to changing rules. In the pre-switch phase, they categorized cards by one dimension (e.g., color). Those who succeeded advanced to the post-switch phase, where sorting was based on a different dimension (e.g., shape). Performance was measured by the number of correct sorts in the post-switch phase, with scores ranging from 0–8 points.

#### Working memory

We used two tasks to assess working memory^45^:

- *Backward digit span:* Children repeated a sequence of numbers in reverse order. The task began with two-digit sequences and increased in difficulty. The highest level achieved (1–5) was recorded as their score.
- *Backward word span:* This task followed the same procedure as the digit span task but used non-semantically related words instead of numbers.

Children completed one set of each working memory task.

### fNIRS measurement

**Fig. 1:**
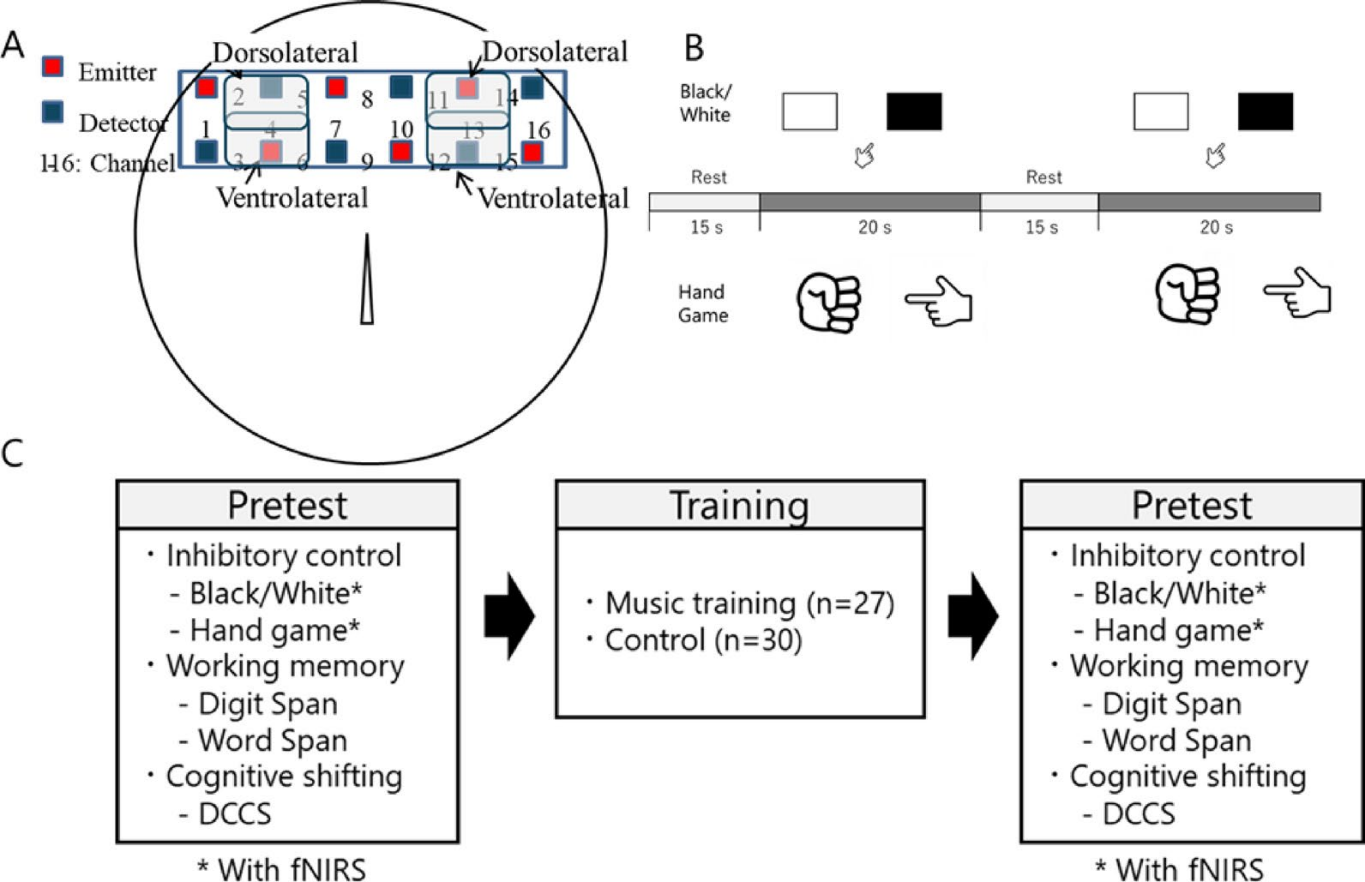
(**A**) fNIRS probe positioning, with each channel consisting of one emitter and one detector optode. Regions of interest (ROIs) include the right and left dorsolateral and ventrolateral prefrontal cortices. (**B**) Experimental procedure for fNIRS measurement. (**C**) Study protocol overview.

We used fNIRS to measure brain activity during the inhibitory control task. The OEG-16 multichannel fNIRS device (Spectratech Inc., Tokyo, Japan) operated at wavelengths of 770 and 840 nm to detect changes in oxygenated (oxy-Hb) and deoxygenated hemoglobin (deoxy-Hb) concentrations. The fNIRS setup consisted of 12 optodes forming 16 channels, positioned bilaterally over the lateral prefrontal regions (Fig. 1A). Each channel comprised an emitter-detector pair spaced 3 cm apart, with a temporal resolution of approximately 666 ms.

During fNIRS measurements, children completed four consecutive sessions each for the Black/White Task and Hand Game in a fixed order (eight sessions in total). Each session comprised a rest phase (15 s) and task phase (20 s) based on a similar study^46^ (Fig. 1B). During the rest phase, children were asked to remain still. During the task phase, the experimenter started a session and gave children inhibitory control tasks. The preceding rest phase was used as a baseline to calculate task-related hemodynamic changes.

Our regions of interest (ROIs)included the dorsolateral and ventrolateral prefrontal cortices, corresponding to F3/4 and F7/8 in the International 10/20 system, respectively. The lower row of the fNIRS probe was positioned between F7 and F8, with the probe’s center at Fpz. Hemoglobin concentration changes were recorded during both the rest and task phases. Data analysis was performed using OEG-16 software (version 3.0) and Python (version 3.6.4).

We calculated the average oxy-Hb and deoxy-Hb changes in each channel during the rest and task phases. To ensure data quality, we first screened for motion artifacts using video recordings and fNIRS data, excluding data points that exceeded three standard deviations from the mean signal amplitude. The raw data underwent bandpass filtering (0.01 Hz -0.5 Hz) and baseline correction via linear fitting. To distinguish functional brain activation from systemic physiological noise, we employed a method based on the linear relationship between oxy-Hb and deoxy-Hb changes, as described in a previous study^47^. This method leverages the known characteristics of NIRS signals, wherein oxy-Hb and deoxy-Hb exhibit a negative correlation during cerebral activity but a positive correlation when reflecting systemic function. We separated the signals into functional and systemic components using this approach, retaining only the functional signals for subsequent analyses. To enhance the signal-to-noise ratio, we averaged data into the right (channels 2, 4, 5) and left (channels 11, 13, 14) channels, as well as right dorsolateral (channels 3, 4, 6) and left ventrolateral (channels 12, 13, 14) prefrontal regions. Channels spanning two regions were weighted equally (0.5) in both areas.

### Procedure

This study was conducted in three distinct stages (Fig. 1C). In the pre-test stage, participants underwent individual assessments lasting approximately 40 min per child. These evaluations included five EF measurements and fNIRS recordings, all conducted in a quiet classroom setting within each participating school. A pair of researchers administered the assessments in a fixed order: the Black/White task (with fNIRS), the Hand Game (with fNIRS), the Backward Digit and Word Span tasks, and the DCCS. The second stage involved the intervention, during which music play activities were integrated into the regular curriculum by the classroom instructor. The intervention consisted of 30 lessons conducted over a 8-week period (3–4 sessions per week, approximately 30 min each). Post-test evaluation repeated the original assessments and maintained the same sequence.

### Statistical analyses

Data analysis was performed using established statistical procedures. For behavioral data, we employed a planned comparison approach rather than omnibus testing to enhance statistical power in evaluating our theoretically-driven hypotheses^48^. Separate paired t-tests were conducted for each group to examine intervention effects. To account for multiple comparisons, we applied Bonferroni correction, setting the significance level at α = 0.025 (0.05/2) for each task. Effect sizes were calculated using Cohen’s d. We also conducted linear mixed modeling for robustness check (see Supplemental analyses). We prioritized the results from our pre-specified planned comparison approach for interpretation.

For fNIRS data analysis, a three-way mixed analysis of variance (ANOVA) was conducted to examine differences based on program (music play vs. control; between-subjects factor), period (pre-test vs. post-test; within-subjects factor), and brain region (right dorsolateral, right ventrolateral, left dorsolateral, or left ventrolateral prefrontal regions; within-subjects factor). Separate ANOVAs were performed for the Black/White task and the Hand Game, with effect sizes reported as partial eta-squared (η^2^). For significant interactions, post hoc tests were conducted using the Bonferroni method to correct for multiple comparisons.

As an exploratory analysis, we examined the relationship between behavioral changes and changes in brain activity before and after the intervention. We calculated correlations between changes in EF task performance (two inhibitory control measures, two working memory measures, and the cognitive shifting task) and oxy-Hb changes in the four target brain regions, separately for the music play and control groups. To account for multiple comparisons, we applied a Bonferroni correction, setting the adjusted alpha level at α = 0.0025 (0.05/20), where 20 represents the number of comparisons (5 tasks × 4 brain regions).

## Results

### Behavioral results

Table 1 presents the behavioral results. Based on our theoretical framework and prior research^46^, we hypothesized that the music group would exhibit greater improvements in EF tasks but the control group would not. To test this hypothesis, we employed a planned comparison approach^48^.

**Table 1:** Mean (SD) of behavioral performance in each task during pre- and post-test.

Task	Music (n = 27)		Control (n = 30)	
	Pre-test	Post-test	Pre-test	Post-test
	M (SD)	M (SD)	M (SD)	M (SD)
Black/white	21.9 (15.6)	31.1 (9.8)	24.3 (13.8)	28.5 (13.0)
Hand game	28.7 (13.2)	34.6 (6.4)	27.72 (13.8)	30.8 (12.7)
DCCS	5.74 (1.87)	6.15 (1.99)	5.60 (1.71)	5.90 (1.95)
Digit span	1.33 (0.62)	1.48 (0.58)	1.37 (0.62)	1.30 (0.54)
Word span	2.07 (0.78)	2.03 (0.59)	1.77 (0.57)	2.07 (1.02)

We conducted separate paired t-tests for each group as part of our planned comparisons to examine the intervention effects. Since two comparisons were planned (pre- to post-test changes in both the music play and control groups) for each task, we applied a Bonferroni correction and set the significance level at α = 0.025 (0.05/2) to control for multiple comparisons.

First, we analyzed the inhibitory control measures. Consistent with our hypothesis, the music play group significantly improved the Black/White task from pre- to post-test (t (26) = − 3.143, *p* = 0.004, Cohen’s d = − 0.605). However, no significant changes were observed in the control group (*t* (29) = − 1.797, *p* = 0.083, Cohen’s d = − 0.328). For the Hand Game, both the music play (*t* (26) = − 2.238, *p* = 0.034, Cohen’s d = − 0.431) and control groups (*t* (29) = − 2.137,* p* = 0.041, Cohen’s d = − 0.390) exhibited a trend of improvement from pre- to post-test, though these results did not survive Bonferroni correction.

Regarding the cognitive shifting task, no significant changes were observed in the DCCS task for either the music play (*t* (26) = − 1.147, *p* = 0.262, Cohen’s d = − 0.221) or control groups (*t* (29) = − 0.866, *p* = 0.393, Cohen’s d = − 0.158).

Finally, we analyzed the working memory measures. Children in the music play group exhibited no significant improvements in either the Digit Span (*t* (26) = − 1.162, *p* = 0.256, Cohen’s d = − 0.224) or Word Span (t (26) = 0.238, *p* = 0.814, Cohen’s d = 0.046) tasks. The control group exhibited a similar pattern, with no significant changes in Digit Span (*t* (29) = 0.701, *p* = 0.489, Cohen’s d = 0.128) or Word Span (*t* (29) = − 1.663, *p* = 0.107, Cohen’s d = − 0.221).

### fNIRS results

**Fig. 2:**
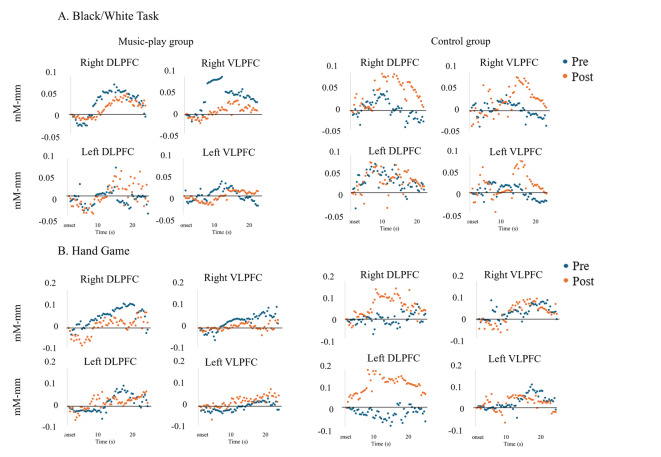
Mean temporal changes in oxygenated hemoglobin concentration within the right and left prefrontal areas during (**A**) the Black/White task and (**B**) the Hand Game. *Abbreviations*: DLPFC – Dorsolateral prefrontal cortex; VLPFC – Ventrolateral prefrontal cortex.

**Fig. 3:**
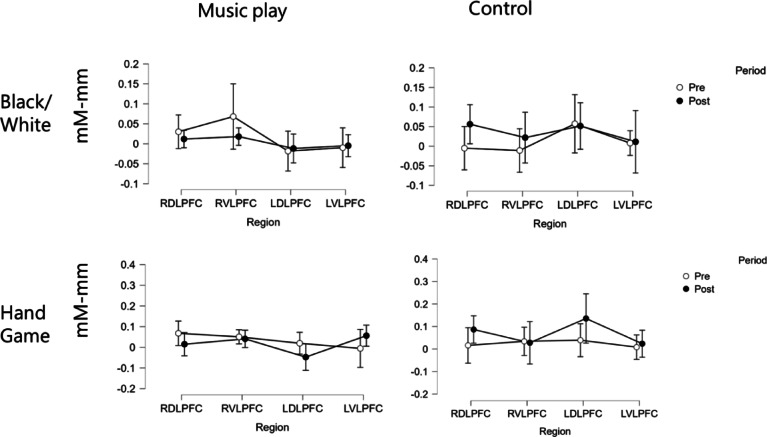
Oxy-Hb changes in each brain region during the pre- and post-test for each task across groups. RDLPFC, RVLPFC, LDLPFC, and LVLPFC represent the right dorsolateral prefrontal cortex, right ventrolateral prefrontal cortex, left dorsolateral prefrontal cortex, and left ventrolateral prefrontal cortex, respectively.

Figure 2 shows the mean temporal changes in oxy-Hb during the Black/White task and the Hand Game. We have conducted additional smoothing applied only for visualization purposes, but all statistical analyses with fNIRS were performed on the 0.01–0.5 Hz filtered data without any additional smoothing. Figure 3 illustrates the mean changes in oxy-Hb concentrations during these tasks. We separately examined whether the lateral prefrontal regions were activated in both tasks. Changes in oxy-Hb were analyzed using a three-way mixed ANOVA, with the program as the between-subject factor and period and brain regions as within-subject factors.

In the Black/White task, no significant main effects were observed for the program (*F*(1, 54) = 0.565, *p* = 0.455, ƞ^2^ = 0.00), period (*F*(1, 54) = 0.069, *p* = 0.793, ƞ^2^ = 0.00), or brain region (*F*(3, 162) = 0.798, *p* = 0.497, ƞ^2^ = 0.00). No significant interactions were observed between program and period (*F*(1, 54) = 1.266, *p* = 0.265, ƞ^2^ = 0.00), or period and region (*F*(3, 162) = 0.222, *p* = 0.881, ƞ^2^ = 0.00). We found a significant interaction between program and region (*F*(3, 162) = 3.322, *p* = 0.021, ƞ^2^ = 0.02), but post-hoc tests using Bonferroni method revealed no significant differences in brain regions between the music play and the control group. Additionally, no significant three-way interaction was observed (*F*(3, 162) = 0.909, *p* = 0.438, ƞ^2^ = 0.00).

In the Hand Game, there were no significant main effects for the program (*F*(1, 54) = 0.736, *p* = 0.395, ƞ^2^ = 0.00), period (*F*(1, 54) = 0.316, *p* = 0.576, ƞ^2^ = 0.01), or brain region (*F*(3, 162) = 0.507, *p* = 0.678, ƞ^2^ = 0.00). However, we observed a significant interaction between program and region (*F*(3, 162) = 3.204, *p* = 0.025, ƞ^2^ = 0.02). No significant interactions were found between program and period (*F*(1, 54) = 1.616, *p* = 0.209, ƞ^2^ = 0.01) or between period and region (*F*(3, 162) = 0.365, *p* = 0.778, ƞ^2^ = 0.01). A trend for three-way interaction was observed (*F*(3, 162) = 2.402, *p* = 0.070, ƞ^2^ = 0.01). Simple interaction analysis revealed a significant interaction between program and region in the post-test (*F*(3, 162) = 4.409, *p* = 0.005, ƞ^2^ = 0.04), but not in the pre-test (*F*(3, 162) = 0.641, *p* = 0.509, ƞ^2^ = 0.01). Post hoc tests using Holm’s method showed that children in the control group exhibited higher activation in the left DLPFC than those in the music play group (*p* = 0.043).

### Additional analyses

We calculated the correlations between changes in EF performance from pre- to post-test (two inhibitory control measures, two working memory measures, and a cognitive shifting task) and oxy-Hb changes in the four target brain regions, separately for the music play and control groups.

**Fig. 4:**
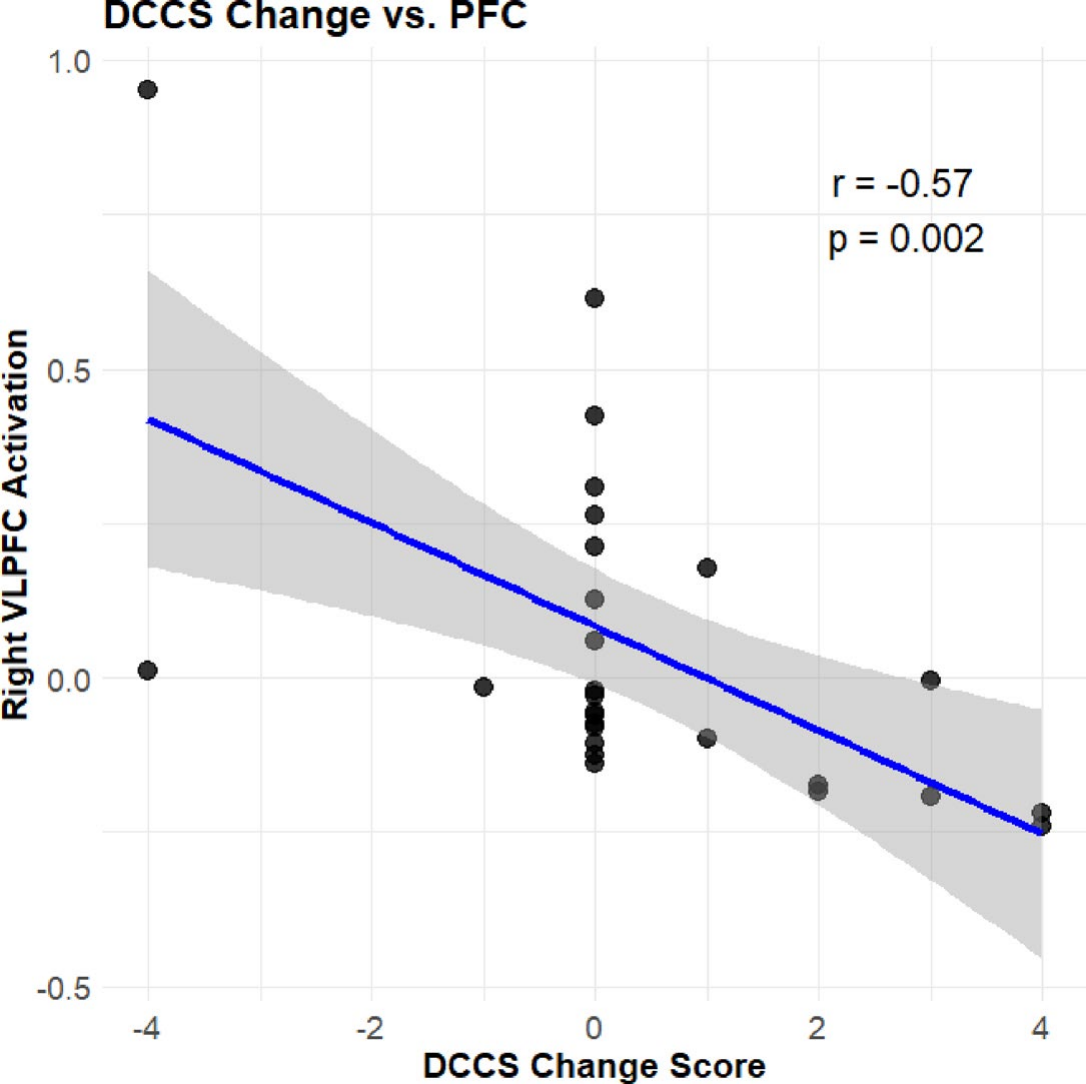
Correlation between changes in DCCS performance and changes in right VLPFC activation during the Hand Game.

In the music play group, performance on the DCCS task was significantly negatively correlated with activation in the right ventrolateral prefrontal region during the Hand Game (r = -0.57, *p* < 0.002) (Fig. 4). No other significant correlations were observed between EF tasks and brain regions in either the Black/White task or the Hand Game. In the control group, no significant correlations were observed between EF tasks and brain activation.

## Discussion

This study examined whether music play influenced EF skills and PFC activity in young children. Participants completed inhibitory control, cognitive shifting, and working memory tasks before and after the intervention. Additionally, PFC activity was assessed during the inhibitory control tasks. During the intervention, children participated in a music play program that included self-directed play activities, movement and expression, and group activities.

Results revealed that children in the music play group showed modest improvements in inhibitory control tasks compared to the control group, whereas no significant improvements were observed in working memory or cognitive shifting tasks. At the neural level, children in the control group exhibited increased activation in the left DLPFC during the post-test compared to the pre-test in inhibitory control tasks. Conversely, children in the music play group showed no significant changes in activation before and after the intervention. Each result has been discussed in detail.

At the behavioral level, the music play intervention demonstrated modest effects on children’s EF skills compared with the control group. Analyses revealed no significant improvements in working memory or cognitive shifting abilities following the music play activities. However, regarding inhibitory control, data suggested modest improvements in the music play group compared to the control group, particularly in the Black/White task. These findings, combined with existing literature, suggest that music play interventions, especially Orff-Schulwerk can enhance inhibitory control more than other EF domains. A previous study^30,31^ reported improvements in both inhibitory control and working memory after music play interventions, although cognitive shifting remained unaffected. Other studies have also corroborated the specific enhancement of inhibitory control through musical training^47^. 

The weaker effects of music interventions in this study—particularly the lack of improvement in working memory—may be due to the small sample size compared to previous studies. A smaller sample size may have limited our statistical power to detect significant behavioral changes in working memory. While conducting pre-and-post neuroimaging studies with young children presents challenges, future research should replicate these findings with larger samples to enhance generalizability.

At the neural level, we observed no significant differences in PFC activity before and after the intervention during the Black/White task, a trend observed in both the music play and control groups. However, in the Hand Game, we observed that children in the control group showed stronger activations in the left DPFFC in the post-test compared to the pre-test. These results were contrary to our expectations, as we hypothesized that the intervention group—who showed improvements in EF skills—would exhibit greater changes in PFC activity than the control group.

These results may be interpreted in terms of neural efficiency. Children in the music play group showed trends toward behavioral improvement in the inhibitory control, yet only the control group exhibited stronger neural activation post-test compared with the pre-test. This suggests that children in the music play group may have developed greater neural efficiency. The observed pattern—behavioral improvement with stable neural activation—points to intervention-specific differences in the development of neural efficiency.

This interpretation is consistent with previous neuroimaging studies in older populations. For example, musical training with instruments has been shown to improve verbal working memory in older adults compared with control groups. Additionally, activation in certain brain regions, such as the right supplementary motor area and bilateral posterior cingulate gyrus, decreased during the task after the intervention^49^, suggesting improved neural efficiency during working memory tasks.

In children, Xie et al.^50^ examined brain activity during EF tasks before and after mindfulness training using multivariate multiscale sample entropy (MMSE) analysis. MMSE measures the complexity or irregularity of brain signals, with higher complexity indicating more variable and potentially less efficient neural processing. Their study found that brain complexity significantly decreased in the prefrontal regions during EF tasks after mindfulness training. This reduction in complexity, coupled with improved behavioral performance, suggests that mindfulness training leads to more efficient and streamlined neural processing. Similarly, since children in the music play group exhibited slight behavioral improvements, they may also have developed more efficient neural processing in the PFC.

This interpretation of neural efficiency was further supported by our exploratory analyses. In the music play group, behavioral changes in the DCCS were negatively correlated with neural changes in the PFC—children who improved their DCCS performance exhibited decreased PFC activation. This trend was not observed in the control group. Although this analysis was exploratory and PFC activity was not directly measured during the DCCS task, the results partially support the neural efficiency hypothesis.

According to Xie et al.^50^, enhanced neural efficiency can be attributed to several mechanisms. First, mindfulness practice helps reduce cognitive load by training sustained attention and minimizing mind-wandering. Second, it promotes emotional regulation and stress reduction, which may decrease unnecessary neural activation. Third, regular mindfulness practice optimizes neural networks through experience-dependent plasticity, allowing individuals to perform cognitive tasks with more streamlined activation patterns. Similar mechanisms may explain how music play enhances neural efficiency in this study.

The Orff-Schulwerk approach to music education, which emphasizes active engagement through exploration and improvisation rather than passive listening, may promote efficient neural processing in several ways. Similar to mindfulness, music play requires sustained attention and emotional regulation during creative expression. Additionally, integrating music, movement, and speech may strengthen neural networks through multimodal engagement. However, key differences exist between mindfulness and music play. The social nature of group music activities demands real-time coordination and adaptation, potentially training children to process complex information more efficiently. Taken together, music play may affect neural efficiency through repeated practice of focused attention, emotional regulation, and flexible adaptation within a playful, developmentally appropriate context.

While our findings did not fully align with our initial hypotheses, this study provides valuable empirical evidence on the impact of music play on EF and PFC development—an area with limited research due to technical and resource constraints. Integrating behavioral and neural measurements in young children, though challenging, offers unique insights into developmental mechanisms. Future research should further investigate these preliminary findings to elucidate the precise mechanisms through which music play influences children’s cognitive and neural development. This could lead to more targeted and effective interventions for enhancing cognitive development in early childhood.

The observed differences in EF may be attributed to the inherent characteristics of the Orff approach. In addition to the self-directedness and social interaction noted in the Introduction, Orff activities fundamentally incorporate physical movement, integrating music with bodily expression. This contrasts with traditional Japanese early childhood education, which primarily emphasizes reading aloud and singing^34,35^. Although physical activity levels were not quantitatively measured in this study, the Orff intervention likely involved greater movement than the control group’s activities. The integration of music with expressive movement is a distinctive feature of the Orff approach that may contribute to its effects on EF development. Future studies should consider measuring physical activity levels to better isolate the specific elements of the Orff approach that influence EF.

Although the present study reveals important findings, it has several limitations. First, due to the onset of the COVID-19 pandemic, we were unable to examine the longitudinal effects of the training. Future studies should incorporate follow-up assessments to determine whether the observed neural changes persist and how they influence developmental trajectories. Second, we did not account for factors such as personality traits, intelligence, and family background, which could influence children’s task performance. While we attempted to mitigate these concerns by randomly assigning participants—ensuring that these factors were theoretically distributed across groups—measuring these variables would have strengthened our study. Additionally, there was some variability in trial timing due to the need for experimenter flexibility when working with young children, which limited the precision of our fNIRS signal analyses. Also, the current control paradigm may not fully isolate the effect of self-directed learning from other factors such as the engaging, varied, and novel nature of the music play program compared to the more passive learning approach in the control condition. Finally, although we observed some changes in brain activation patterns, the group differences in the prefrontal regions were rather small. Future studies should investigate other brain regions (e.g., the parietal cortex) and employ multiple neuroimaging methods (e.g., fNIRS, EEG) to provide more comprehensive insights into the underlying neural processes. Nonetheless, our findings offer promising initial evidence of the potential for music play to influence neural processing in young children.

## Electronic supplementary material

Below is the link to the electronic supplementary material.

Supplementary Material 1

## Data Availability

All experimental data are available from 10.6084/m9.figshare.28057613.v2.
