# Supplementary material for: Self-directed music play to improve executive function in young children using NIRS
Source: Sci Rep. 2025 Jul 22;15:26608. doi: 10.1038/s41598-025-10984-1 (PMC12284005; doi:10.1038/s41598-025-10984-1)
Supplement: Supplementary file 1 — Supplementary Material 1 [file 41598_2025_10984_MOESM1_ESM.docx]

Supplemental Analysis

To examine the effects of Group (music play vs. control) and Time (pre-test vs. post-test) on EF scores, we conducted linear mixed model (LMM) analyses using R software (version 4.2.3) with the lme4 package (Bates et al., 2015). EF scores served as the dependent variable, with Group and Time and their interaction as fixed effects. To account for individual differences among participants, participant ID was included as a random effect.

Inhibitory Control

We found a significant main effect of Time (b = 9.22, SE = 2.72, p = .001), but not Group (b = 2.42, SE = 3.51, p = .493) in the Black/White task. No significant interaction was observed (b = -4.96, SE = 3.74, p = .191). Similar results were observed in the Hand Game, showing only a main effect of Time (b = 5.82, SE = 2.19, p = .010), but not Group (b = -1.51, SE = 3.18, p = .636), and no interaction between variables (b = -2.28, SE = 3.02, p = .453).

Cognitive Shifting

No significant Group (b = -0.14, SE = 0.50, p = .779), Time (b = 0.41, SE = 0.36, p = .263) effects, nor interaction between variables (b = -0.11, SE = 0.50, p = .830) were observed in the DCCS task.

Working Memory

We found no significant effects of Group (b = 0.03, SE = 0.16, p = .831), Time (b = 0.15, SE = 0.11, p = .199), or their interaction (b = -0.21, SE = 0.16, p = .177) on the Digit Span task. The same pattern was observed for the Word Span task (Group: b = -0.30, SE = 0.20, p = .132; Time: b = -0.03, SE = 0.17, p = .833; interaction: b = 0.33, SE = 0.24, p = .167).

The results were generally consistent with our planned comparisons, although we note that we did not find a significant interaction between Group and Time in the LMM analysis of the Black/White task. Despite this discrepancy between the LMM results and our a priori planned comparisons, we prioritized the results from our pre-specified planned comparison approach for interpretation. However, we acknowledge that this inconsistency somewhat weakens the strength of our conclusions regarding the interaction effect in inhibitory control tasks.
